# Outcome Determinants of Patients with Traumatic Pelvic Fractures: A Cohort Study in a Level I Trauma Center in Southern Iran

**DOI:** 10.5704/MOJ.1711.012

**Published:** 2017-11

**Authors:** S Paydar, M Chaabi, M Akhavan, Z Ghahramani, M Dehghankhalili

**Affiliations:** Trauma Research Center, Shiraz University of Medical Sciences, Shiraz, Iran; * Department of General Surgery, Shiraz University of Medical Sciences, Shiraz, Iran; ** General Practitioner, Shiraz Univerisity of Medical Sciences, Shiraz, Iran

**Keywords:** pelvic fracture, outcome, mortality, surgery, prognosis

## Abstract

Pelvic fracture is a result of devastating injuries and is usually encountered in conjunction with other life-threatening injuries. The aim of the current study was to determine the outcome determinants of patients with pelvic fractures referred to a large trauma center in southern Iran. This retrospective cross-sectional study was conducted in a level I trauma center over a period of three years from 2012 to 2015. We included all patients with pelvic fractures whose medical records had sufficient data. Data were compared between good condition and poor conditions. A total of 327 patients with mean age of 40.1 ± 19.7 years were included. Poor condition was defined as being associated with higher heart rate (p=0.002), lower systolic blood pressure (p<0.001), lower diastolic blood pressure (p=0.002) lower Glasgow Coma Scale (GCS) on admission (p<0.001) and higher Injury Severity Score (ISS) (p<0.001). Those with poor conditions had significantly higher admission to ICU (p<0.001), higher rate of surgical interventions (p<0.001) and higher mortality (p<0.001). The hospital length of stay (p<0.001) and ICU length of stay (p=0.025) were also longer in those with poor condition. Lower hemoglobin, lower pH, higher heart rate, lower systolic blood pressure, lower GCS on admission and higher ISS were important outcome determinants of traumatic pelvic fractures.

## Introduction

Pelvic fractures are relatively uncommon, with a reported incidence of only 2-8% of all fractures, but in multiple trauma patients, the frequency can rise up to more than 25%^1,2^. Pelvic fractures continue to represent a challenge in terms of life-threat and functional outcome. Despite advances in management during the past decade, mortality remains significantly high, with mortality rates ranging between 10 and 16%^3,4^. The risk of hemorrhage makes pelvic fractures the most serious skeletal injury resulting in substantial mortality that ranges from 5 to 50% in the literature and is dependent not only on the type of pelvic ring fracture but also on the severity of associated injuries involving the abdomen, chest, and central nervous system^5-7^.

To be useful, any predictive system must use only information that is available early in the patient’s course of management. Age, fracture pattern, systolic blood pressure on arrival, base deficit, and the Revised Trauma Score (RTS) are all pieces of information that are available to the clinician quickly after patient presentation^8,9^. Several studies have demonstrated that age, severity of injury determined by injury severity score (ISS), requiring more blood transfusion and co-injuries are among the most important determinants of outcome in patients with traumatic pelvic fractures^10,11^.

Although the outcome determinants of the pelvic fractures have been previously described, data from the Iranian population is scarce in the literature. The aim of the current study was to determine the outcome determinants of patients with traumatic pelvic fractures referred to an Iranian large level I trauma center.

## Materials and Methods

This retrospective cross-sectional study was conducted in Shahid Rajaee (Emtiaz) Trauma Hospital (a level I referral center for trauma patients over 14 years of age at Fars Province, affiliated to Shiraz University of Medical Sciences, Shiraz, Iran) over a period of three years from March 2012 to February 2015. This referral trauma hospital is a 200-bed government academic adult trauma center of Shiraz. The study protocol was approved by the institutional review board (IRB) and medical ethics committee of Shiraz University of Medical Science. As this was a retrospective study, no informed written consent was required. The data was obtained from the patients’ medical records. We included all multiple trauma patients with pelvic fractures (stable or unstable) aged more than 14 years who were injured in motor vehicle accidents or fall. We excluded patients with penetrating pelvic trauma, age less than 14 years and those with incomplete document. Those who passed away due to severity of injuries during the primary surgery were also excluded. All the patients were managed according to the ATLS guidelines (primary and secondary surgery). After stabilization of the hemodynamic parameters, orthopaedic management was instituted and retroperitoneal packing and pelvic fixation were performed if needed.

The variables examined included: age, gender, mode of transportation and duration and care provided, mechanism of trauma (motor vehicles or falling down), initial vital signs (blood pressure, heart rate, respiratory rate, O2 saturation), laboratory data within 24 hours of admission (CBC, BUN, creatinine, and electrolytes), co-injuries (head trauma, thoracic trauma, abdominal trauma), need for blood products transfusion and patients’ outcomes. The patients’ outcomes included: transfer to ward, transfer to ICU, transfer to operation room, and early and late in-hospital mortality. Mortality in the first 24 hours was considered as early hospital mortality, and late hospital mortality was if the patient had either a hospital result of death after first 24 hours. Sociodemographic information were obtained through interview with patients (or persons accompanying patients if they were too ill to impart this information) and laboratory, clinical and other information obtained from the patient’s medical records. Patients were further categorized as good condition and poor condition. Those with poor condition were those who had received more than two units packed red blood cells, had undergone operation for pre-peritoneal pelvic pack and external pelvic fixation less than 24 hours of admission and those who had died. Even if only one of these criteria was present, the case was defined as poor condition. The clinical, laboratory and types of fractures were further compared between these two study groups to identify the determinants of outcome in patients with pelvic fractures admitted to our center.

Data was analyzed using statistical package for social sciences version 17 (SPSS Inc., Chicago, IL, USA). Normality of data was tested by Kolmogorov–Smirnov test and the frequency distributions (histograms). All data were expressed as mean ± SD or number and percentage as needed. Pearson’s chi-square or Fisher’s exact test was used to compare the difference of proportions. Multivariate analysis was performed by multiple logistic regression by the method “backward stepwise” with results expressed by Odds Ratios and their confidence intervals (CI) of 95% to identify predictors of hospital mortality factors. Independent variables were included in the multivariate logistic regression model with p up to 0.25. A 2-sided p-value of less than 0.05 was considered significant.

## Results

We included a total of 327 patients with pelvic fractures and with multiple trauma referred to our center during the study period. The mean age of the patients was 40.1 ± 19.7 (range: 15 to 90) years. There were 218 (66.7%) men and 109 (33.3%) women. We found that superior and inferior pubic rami fractures were the most common types of pelvic fracture. Femur head fractures were the least common. The baseline characteristics of the patients are summarized in Table I.

**Table I:** The baseline characteristics of 327 trauma patients with pelvic fractures referred to our center during the study period

Variable	Value
Age (years)	40.1 ± 19.7
Gender	
Men (%)	218 (66.6%)
Women (%)	109 (33.3%)
Mechanism of injury	
Motor-vehicle accident (%)	258 (78.9)
Fall (%)	53 (16.2%)
Assault (%)	3 (0.9%)
Sharp object (%)	1 (0.3%)
Gunshot (%)	1 (0.3%)
Shotgun (%)	1 (0.3%)
Stab wound (%)	1 (0.3%)
Others (%)	7 (2.1%)
Heart rate (per min)	97.2 ± 53.6
Respiratory rate (per min)	18.5 ± 9.46
Systolic blood pressure (mmHg)	124.89 ± 24.9
Diastolic blood pressure (mmHg)	77.06 ± 16.31
O2 saturation (%)	92.8 ± 6.43
GCS	13.94 ± 2.52
Mile (%)	272 (83.2%)
Moderate (%)	30 (9.2%)
Severe (%)	25 (7.6%)
ISS	11.79 ± 7.52
Airway	
Patent	273 (83.5%)
Intubated	54 (16.5%)
Fractures	
Right iliac bone (%)	42 (12.8%)
Right superior pubic ramus (%)	131 (40.1%)
Right inferior pubic ramus (%)	134 (41.0%)
Right sacral bone (%)	32 (9.8%)
Right ischial bone (%)	7 (2.1%)
Right acetabulum (%)	25 (7.6%)
Right hip joint (%)	16 (4.9%)
Right femoral neck (%)	3 (0.9%)
Right greater trochanter (%)	6 (1.8%)
Right lesser trochanter (%)	4 (1.2%)
Right diastasis of sacroiliac joint (%)	26 (8.0%)
Left iliac bone (%)	44 (13.5%)
Left superior pubic ramus (%)	136 (41.6%)
Left inferior pubic ramus (%)	139 (42.5%)
Left sacral bone (%)	29 (8.9%)
Left ischial bone (%)	20 (6.1%)
Left acetabulum (%)	29 (8.9%)
Left hip joint (%)	9 (2.8%)
Left femoral neck (%)	7 (2.1%)
Left greater trochanter (%)	6 (1.8%)
Left lesser trochanter (%)	5 (1.5%)
Left diastasis of sacroiliac joint (%)	23 (7.0%)
Diastasis of symphysis pubis (%)	45 (13.8%)

GCS: Glasgow Coma Scale; ISS: Injury Severity Score

The baseline, para-clinical, clinical and laboratory findings were compared between those with good and poor conditions in order to determine the outcome determinants of pelvic fractures in our series. We found that poor condition was associated with higher heart rate (p=0.002), lower systolic blood pressure (p<0.001), lower diastolic blood pressure (p=0.002) lower GCS on admission (p<0.001) and higher ISS (p<0.001). The results are summarized in Table II. We found that poor condition was associated with lower pH (p=0.002), lower base excess (p<0.001), lower hemoglobin level (p=0.001), more packed red cell transfusion (p<0.001) and larger volume of infused intravenous fluid (p=0.020).

**Table II:** Comparison of the baseline demographic, clinical and laboratory characteristics between those with good and poor conditions

	Good condition (n=237)	Poor condition (n=90)	p-Value
Age (years)	40.2 ± 19.8	39.7 ± 19.5	0.832
Gender			
Men (%)	153 (64.6%)	65 (72.2%)	0.118
Women (%)	84 (35.4%)	25 (27.8%)	
Heart rate (per min)	92.1 ± 18.1	110.7 ± 96.9	0.002
Respiratory rate (per min)	18.2 ± 7.8	19.3 ± 12.8	0.356
SBP (mmHg)	128.1 ± 24.2	116.6 ± 25.2	<0.001
DBP (mmHg)	78.8 ± 14.7	72.5 ± 19.2	0.002
O2 saturation (%)	93.1 ± 6.6	91.9 ± 5.9	0.165
GCS	14.28 ± 2.06	13.07 ± 3.23	<0.001
Mile (%)	208 (87.5%)	64 (71.1%)	
Moderate (%)	16 (6.9%)	14 (15.6%)	0.002
Severe (%)	13 (5.6%)	12 (13.3%)	
ISS 10.64 ± 3.69	14.83 ± 12.56	<0.001	
pH 7.37 ± 0.07	7.32 ± 0.10	0.002	
HCO_3-_ (mEq/L)	19.99 ± 9.61	18.92 ± 6.62	0.371
Base excess (mEq/L)	0.84 ± 8.37	−3.63 ± 7.41	<0.001
PT (sec)	13.70 ± 2.65	14.13 ± 1.89	0.192
PTT (sec)	35.35 ± 9.21	38.08 ± 17.63	0.108
INR 1.33 ± 0.83	1.35 ± 0.49	0.819	
Hemoglobin (mg/dL)	12.86 ± 2.07	11.94 ± 2.48	0.001
Fibrinogen (µg/L)	14.65 ± 13.32	13.32 ± 9.06	0.636
Transfused packed RBC	1.01 ± 0.1	4.11 ± 2.75	<0.001
Infused IV fluid (mL)	1130.24 ± 422.3	1379.31 ± 561.4	0.020

DBP: Diastolic blood pressure; IV: Intravenous; INR: International normalizing ratio; PT: Prothrombin time; PTT: Partial thromboplastin time; RBC: Red Blood cell; SBP: Systolic blood pressure

We compared the type of pelvic fracture between those with good and poor conditions. We found that poor condition was associated with left iliac bone fracture (p=0.018), diastasis of left sacroiliac joint (p=0.030) and diastasis of symphysis pubis (p<0.001). None of the other types of fracture was associated with the outcome of the patients (Table III). The long bone fractures were not associated with outcome of patients with pelvic fractures. However, those with poor condition had significantly higher admission to ICU (p<0.001) and higher rate of surgical interventions (p<0.001), longer hospital length of stay (p<0.001) and ICU length of stay (p=0.025). The results are summarized in Table VI. The logistic regression model revealed that heart rate (p=0.007), systolic blood pressure (p=0.046), ISS (p<0.001) and diastasis of the symphysis pubis (p=0.003) remained significant determinants of outcome after eliminating the role of co-existing risk factors.

**Table III:** The type of pelvic fracture in 327 trauma patients with pelvic fractures referred to our center during the study period

Variable	Good condition (n=237)	Poor condition (n=90)	p-Value
Right iliac bone (%)	26 (11.0%)	16 (17.8%)	0.137
Right superior pubic ramus (%)	92 (38.8%)	39 (43.3%)	0.528
Right inferior pubic ramus (%)	90 (38.0%)	44 (48.9%)	0.079
Right sacral bone (%)	22 (9.3%)	10 (11.1%)	0.677
Right ischial bone (%)	5 (2.1%)	2 (2.2%)	0.998
Right acetabulum (%)	16 (6.8%)	9 (10.0%)	0.353
Right hip joint (%)	14 (5.9%)	2 (2.2%)	0.252
Right femoral neck (%)	1 (0.4%)	2 (2.2%)	0.185
Right greater trochanter (%)	4 (1.7%)	2 (2.2%)	0.669
Right lesser trochanter (%)	3 (1.3%)	1 (1.1%)	0.998
Right diastasis of sacroiliac joint (%)	18 (7.6%)	8 (8.9%)	0.655
Left iliac bone (%)	25 (10.5%)	19 (21.1%)	0.018
Left superior pubic ramus (%)	93 (39.2%)	43 (48.8%)	0.169
Left inferior pubic ramus (%)	95 (40.1%)	44 (48.9%)	0.169
Left sacral bone (%)	18 (7.6%)	11 (12.2%)	0.196
Left ischial bone (%)	16 (6.8%)	4 (4.4%)	0.607
Left acetabulum (%)	16 (6.8%)	13 (14.4%)	0.047
Left hip joint (%)	4 (1.7%)	5 (5.6%)	0.121
Left femoral neck (%)	6 (2.5%)	1 (1.1%)	0.678
Left greater trochanter (%)	4 (1.7%)	2 (2.2%)	0.669
Left lesser trochanter (%)	3 (1.3%)	2 (2.2%)	0.618
Left diastasis of sacroiliac joint (%)	12 (5.1%)	11 (12.2%)	0.030
Diastasis of symphysis pubis (%)	21 (8.9%)	24 (26.7%)	<0.001

## Discussion

In the current study, we included a large series of trauma patients with pelvic fractures referred to a large trauma center in southern Iran. We found that poor condition pelvic fractures are associated with higher heart rate, lower systolic and diastolic blood pressure, lower GCS on admission and higher ISS. On admission lower pH, lower base excess, and lower hemoglobin level were among the poor outcome determinants of pelvic fractures. Left iliac bone fracture, left diastasis of sacroiliac joint and diastasis of symphysis pubis were important determinants of poor outcome in patients with pelvic fracture. These group of patients had higher admission to ICU and higher rate of surgical interventions. The hospital and ICU length of stay were also longer in those with poor condition. This point should be taken into consideration in those with poor condition with severe injury requiring initial transfusion of more than 2 units packed cells, emergency retroperitoneal packing or external fixation. Thus, the predicting factors are affected by these inclusion criteria. However, we defined these criteria in order to determine some predictors of the outcome in these patients. We propose that this definition of poor condition could be used in the emergency room for initial classification of the patients.

**Table IV:** Comparison of outcome measures between patients with long bone fractures and pelvic fractures with good and poor conditions

Variable	Good condition (n=237)	Poor condition (n=90)	p-value
One fracture (%)	53 (22.4%)	20 (22.2%)	0.997
Two unilateral fractures (%)	66 (27.8%)	18 (20.0%)	0.159
Two bilateral fractures (%)	9 (3.8%)	5 (5.6%)	0.542
≥3 Unilateral fractures (%)	43 (18.1%)	13 (14.4%)	0.512
≥3 Bilateral fractures (%)	66 (27.8%)	34 (37.8%)	0.106
Outcome			
Operation (%)	32 (13.5%)	28 (31.1%)	<0.001
Admission to ICU (%)	57 (24.1%)	42 (46.7%)	<0.001
ICU LOS (days)	2.75 ± 7.9	5.01 ± 8.41	0.025
Hospital LOS (days)	10.13 ± 11.58	19.78 ± 18.2	<0.001
Mortality (%)	18 (7.59%)	34 (37.7%)	<0.001

LOS: Length of stay

**Table V:** The results of multivariate logistic regression model determining the predictive mortality in 327 patients with traumatic pelvic fractures after adjusting for independent variables (age, gender and mechanism of injury)

	p-Value	Odds ratio (95% CI)
Heart rate (per min)	0.103	1.011 (0.998-1.567)
SBP (mmHg)	0.046	0.988 (0.976-1.017)
GCS	0.007	0.871 (0.789-0.922)
ISS	<0.001	1.137 (1.061-1.263)
Base excess (mEq/L)	0.017	3.153 (1.227-4.863)
Diastasis of symphysis pubisis	0.003	0.336 (0.162-0.363)

GCS: Glasgow coma scale; ISS: Injury severity score; SBP: Systolic blood pressure

Pelvic fractures are still among the most devastating musculoskeletal injuries being associated with high mortality and morbidity, despite advances in knowledge regarding underlying pathophysiology and enhancements in the surgical techniques^12,13^. The mortality rate, even for uncomplicated cases, remains among the highest for any musculoskeletal injury^13^ and complications following treatment have been found to occur frequently, often resulting in inferior outcomes^14^. Improved understanding regarding patient-based characteristics with predictable risk of complications following pelvic trauma and mortality could enhance patient care. Risk factors that are potentially modifiable, such as perioperative glucose control, the timing of surgical intervention and blood pressure management could have a direct impact on post-injury management, while appreciation of the effect that factors such as age, mechanism of injury and medical co-morbidities which influence outcome could be used in informed counselling, shared decision making and expectation of management^15^.

The incidence of pelvic fractures has been reported to be approximately 3% of skeletal injuries, with associated mortality rates ranging between 10 and 16%^16,17^. However, open pelvic fractures are rare and encompass only 2-4% of all pelvic fractures^18^. Pelvic fractures are among the most severe injuries following trauma being associated with high mortality and morbidity^16^. These injuries usually result from high energy trauma and involve the application of either direct or transmitted forces^3^. Adult patients sustaining pelvic fractures are usually young males with multiple injuries due to road traffic accidents. In the current study, there was a male predominance and motor-vehicle accidents was the most common cause followed by fall from height. This is in accordance with previous studies^15,19^. Sen *et al*^20^ reported that the most common mechanism of injury in patients with unstable pelvic fractures was automobile and pedestrian collision, accounting for 36% of cases, while motor vehicle collision occurred in 30% of cases. In the study conducted by Moreno *et al* ^21^ and Dalal *et al*^22^, 40% and 57% of unstable pelvic fractures were due to motor vehicle collision respectively. It has been observed that motor vehicle collision cases are associated with greater incidence of shock, associated injuries and mortality; however, we did not find any relationship between mechanism of injury and the outcome of the patients. In the study by Dalal *et al*^22^ lateral compression injuries were commonly associated with motor vehicle collision. Anteroposterior compression Type 3 injury is common in automobile pedestrian collision and vertical shear common in fall from height. However, Sen *et al*^20^ did not find such relationship between fracture types and mechanism of injury. In the current study, we did not evaluate any such relationship.

Absence or presence of associated injuries to the head, chest or abdomen is the main determinant of patient’s survival^20^. Arroyo *et al*^15^ reported that increasing age, shock, time to procedure, ISS, and GCS were predictive of mortality in patient with pelvic fractures. They also reported that cardiac events were found to be influenced by obesity, diabetes, ISS, GCS, age, and trauma mechanism^15^. Injuries caused by mechanisms other than blunt trauma, ISS, GCS, age, medical co-morbidities, shock, and time to procedure were associated with infection^15^.

Limited prognostic studies are available that identify specific risk factors for death after pelvic trauma^6,15,20,23^. Previous reports are generally concordant with our findings. Arroyo *et al*^15^ demonstrated that age, medical co-morbidities, GCS and ISS are predictors of outcome in patients with pelvic fractures. However, we demonstrated that age and gender were not among the predictors of outcome of patients with pelvic fractures. This could be due to the fact that in our series, most of the victims were young men involved in motor-vehicle accidents. We showed that diastasis of the symphysis pubis was among the most important indicators of the outcome in patients with pelvic fractures. This is an interesting finding while previous studies have not reported such an association. This finding could be explained due to the fact that diastasis of symphysis pubis is among the most severe fracture types of the pelvic being associated with high emergency trauma resulting in concomitant injuries to the chest, abdomen and brain.

Arroyo *et al*^15^ also determined the risk factors of complications such as infection, cardiac events and thromboembolism in patients with pelvic fractures. They reported that cardiac events were associated with obesity, diabetes, ISS, GCS, age, and trauma mechanism. Injuries caused by mechanisms other than blunt trauma, shock, age, ISS, GCS and medical co-morbidities, and time to procedure were associated with infection^24^. In addition, Arroyo *et al*^15^ reported respiratory disease, male sex, medical comorbidities, and time to intervention as another risk factors of DVT in patients with pelvic fractures. They also demonstrated the effects of age, obesity, and diabetes on cardiac events post-injury. In the current study, we did not evaluate these complications and their determinants. We categorized the patients based on the severity of the injury defined as the need for more than 2 units of packed red blood cells, need for early surgical intervention and mortality. Thus, we can only comment on the risk factors of outcome in patients with pelvic fractures.

Increased patient age and ISS have been reported to be predictive of mortality following pelvic injury^15,20,25,26^. O’Brien *et al*^25^ maintained that individuals of age 55 and older were at elevated risk of death after pelvic fracture, and Gabbe and colleagues reported similar findings^6^. Arroyo *et al*^15^ found that when compared to those 18-59, patients aged 71 and older showed a significantly increased risk of mortality, with those in the range 81-90 years carried a remarkable risk nearly 24 times that of the referent. As most of our patients were young men injured in motor-vehicle accidents, we could not find any association between age and the outcome of the patients with pelvic fractures.

We note some limitation to our study. First, this was retrospective study determining and recording the variable based on the medical records which often had incomplete data and information. Thus, we had to eliminate a huge number of the patients due to incomplete medical records. Second, we did not record the risk factors and associated complications and thus could not comment on their effects following the pelvic fractures. Taking all these together, this is the only study from Iran that reports the determinants of the outcome in trauma patients with pelvic fractures.

## Conclusion

We found that poor condition pelvic fracture is associated with higher heart rate, lower systolic blood pressure, lower GCS on admission and higher ISS. It was also associated with lower pH and hemoglobin level. Diastasis of symphysis pubis is among the most important indicators of the outcome in patients with traumatic fracture of pelvis. Those with poor condition pelvic fracture have longer duration of hospital and ICU stay.
